# Impact of simulation-based training in addition to theoretical training versus theoretical training of nurses alone in the occurrence of adverse events related to arteriovenous fistula puncture in chronic hemodialysis patients: study for a cluster randomized controlled trial (SIMFAV2)

**DOI:** 10.1186/s13063-023-07513-8

**Published:** 2023-08-07

**Authors:** Erwan Guillouet, Clémence Tomadesso, Bibiana Acevedo Flores, Patrick Henri, Antoine Lanot, Rémy Morello, Sonia Guillouet

**Affiliations:** 1 NorSimS Simulation Center, Caen University Hospital, Caen, France; 2 Clinical Research Department, Caen University Hospital, Caen, France; 3 Department of Nephrology, Caen University Hospital, Caen, France; 4 Health Training and Research Center, Caen University, Caen, France

**Keywords:** Adverse events, Arteriovenous fistula, Nurse, Simulation, Simulation-based training

## Abstract

**Background:**

The technique of arteriovenous fistula (AVF) puncture is currently taught by colleagues within hemodialysis units. Even if the main principles of the technique are well known and common to all hemodialysis units, strong rationales are still missing to standardize fine practices such as the relative position of the needles, the angle of the needle at puncture, and the position of the bevel at the time of puncture and after the needle is in the vascular lumen.

**Methods:**

We are conducting a prospective, comparative, center-randomized, multicenter study involving 8 hemodialysis centers. The primary objective is to compare the number of adverse events related to AVF puncture between a group receiving theoretical training plus simulation-based training (4 centers) and a group receiving only theoretical training (4 centers). The study will include all adult patients who are scheduled to have an AVF puncture performed by a hemodialysis-trained nurse during a scheduled chronic dialysis session.

**Discussion:**

We hypothesize that a training program for nurses on the AVF approach in procedural simulation versus theoretical input alone would decrease the adverse events related to AVF punctures and would be beneficial for the patient. This study is innovative for several reasons. First, simulation-based training in continuing education among professionals is not widely used. Furthermore, training allows for the standardization of practices within the team, both technically and relationally.

**Trial registration:**

ClinicalTrials.gov NCT05302505. Registered on March 17, 2022.

## Administrative information

**Table:** 

Title {1}	Impact of simulation-based training in addition to theoretical training versus theoretical training alone on the occurrence of adverse events related to arteriovenous puncture in chronic hemodialysis patients. SIMFAV2: study for a cluster randomized trial
Trial registration {2a and 2b}	NCT05302505 https://clinicaltrials.gov/ct2/show/NCT05302505 Date registered: March 17^th^, 2022
Protocol version {3}	2.0, January 25^th^, 2022
Funding {4}	This research is funded by the Hospital Program of Nursing and Paramedical Research planned by the French Department of Health. The study is coordinated by the University Hospital of Caen (Unique Protocol ID: 2021-A01813-38)
Author details {5a}	^1^NorSimS simulation center, Caen University Hospital, Caen, France ^2^Clinical Research Department, Caen University Hospital, Caen, France ^3^Department of Nephrology, Caen University Hospital, Caen, France ^4^Health training and research center, Caen University, Caen, France
Name and contact information for the trial sponsor {5b}	Trial Sponsor: Caen University Hospital of Caen Email: drci-secretariat@chu-caen.fr
Role of sponsor {5c}	Management, analysis, and interpretation of data

## Introduction

### Background and rationale {6a}

Chronic renal failure refers to a decrease in the purification function of the kidneys. When chronic renal failure reaches the end stage, only the implementation of renal replacement therapy allows the affected subjects to stay alive. Three types of replacement therapy are offered to patients with end-stage renal disease: transplantation, extrarenal purification by peritoneal dialysis, and extrarenal purification by hemodialysis (HD).

### Hemodialysis

Hemodialysis is the first modality of treatment in France [1]. It is a technique of purification of uremic toxins accumulated in the blood of patients via extracorporeal circulation and a semipermeable membrane. This treatment requires specific technical skills from the nephrology service providers who are in charge of it. There are four basic elements to consider when performing hemodialysis treatment: the vascular approach, the extracorporeal circulation circuit in which the patient’s blood circulates, the artificial kidney or dialyzer, and the HD machine. The quality of the treatment depends largely on the quantity of blood that can be treated during the hemodialysis session and therefore on the quality of the vascular approach from which the blood is extracted and returned to the patient. There are two types of vascular access, central venous catheter and arteriovenous fistula (AVF), which is the preferred type of access.

### Arteriovenous fistula

AVF is a communication between an artery and a superficial vein, which is created surgically, and after a maturation period of at least 6 weeks, it allows us to obtain a portion of the superficial vein of good caliber in which the blood flow is sufficiently important to allow the realization of hemodialysis. The AVF must be punctured by a nurse at each session with two large gauge needles to allow sufficient blood flow. This step, which is repeated more than 300 times a year per patient, is crucial to the success of the treatment. The quality of its execution determines the success of the treatment [2]. This procedure requires specific skills [3] to prevent the many complications that can arise from incorrect positioning of the needle in the AVF lumen, pain, hematoma at the puncture site, false aneurysm or pulsatile hematoma, transfixing puncture of the vein, infection, scarring, and even necrosis at the puncture site with a risk of unpredictable AVF rupture and cataclysmic acute bleeding [4].

The psychological aspect must be taken into account, as difficult or even painful punctures often cause apprehension in patients, even real anxiety, and can be a source of deterioration in quality of life.

Finally, the quality of the AVF puncture also impacts the quality of the treatment [5]. In the case of malposition of the needles in the vascular lumen, the blood flow and thus the quantity of blood treated can be greatly reduced. A more severe puncture complication may render the vascular approach unusable for several days [6].

The population of subjects treated in hemodialysis is made up of increasingly older patients with vascular comorbidities and often diabetes. All these factors negatively influence the quality of the vessels for AVF preparation and contribute to increasing the risk of complications related to AVF puncture.

### AVF puncture technique

Currently, the technique of AVF puncture is taught by colleagues within hemodialysis departments. The contribution of a training program on the approach to AVF in procedural simulation seems to be relevant [7]. Although the main principles of the technique are well known and common to all hemodialysis units, strong rationales are still lacking to standardize fine practices such as the relative position of the needles, the angle of the needle at puncture, and the position of the bevel at the time of puncture and after the needle is in the vascular lumen. This technique has not yet been precisely protocolized. The empirical observations made in the centers show complications of AVF puncture. These could probably be avoided by improving the quality of the puncture [8].

Thus, the standardization of the AVF puncture technique could be part of a process of optimization of practices and continuous improvement of care [9].

Procedural simulation appears to be an interesting tool to ensure the conceptualization of the AVF approach.

### Procedural simulation

The term Simulation in Health refers to the use of hardware (such as a mannequin or procedural simulator), virtual reality, or a standardized patient, to replicate care situations or environments, for the purpose of teaching diagnostic and therapeutic procedures and rehearsing processes, medical concepts, or decision-making by a health care professional or team of professionals. According to the French Health Authority, health care simulation is intended for all health care professionals and allows training in procedures, the management of situations, acquiring and updating technical and nontechnical knowledge and skills, and analyzing professional practices with debriefings [10].

Simulation-based teaching allows students to learn from experience, develop individual skills [11], and know and apply procedures in continuing education. However, it is not widely used, although it could be used to update knowledge of procedures and to harmonize practices that have been transmitted from one nurse to another. It allows professionals to adapt to new practices and recommendations and to acquire new knowledge and skills to serve the patient [12]. Simulation-based learning can also accompany other forms of simulation, such as training AVF on a simulated patient wearing a procedural simulator (hybrid simulation) [13].

The notion of performance implies the concept of evaluation in simulation. This can be based on the performance of the trainee but also on the evaluation of the training itself, according to the Kirkpatrick model [14, 15], the reference model for evaluation. Level 1 measures the degree of satisfaction of the learner. Level 2 is a pedagogical evaluation that measures what the learner has learned. Level 3 assesses how the learner modifies a procedure in a given scenario. The first three levels are directly related to the impact on the learner. The last level measures the direct benefit to the patient. It looks at the benefits of the training in the real world: what the changes did for the patient.

Studies have been carried out on the impact of simulation training on performance or on the reduction of iatrogeny, particularly during the continuing education of professionals. In the study by Mac Master et al. [16] on the “removal of fish hooks” in emergency departments, it was shown that after a training program including simulation, the hooks were removed without complications. Regarding anesthesia, Bruppacher et al. [17] showed that training through simulation versus interactive seminars increased the care performance of senior physicians at the end of extracorporeal circulation procedures in the operating room. Finally, in a recent study, Ajmi et al. [18] showed that after a hybrid simulation training program on the management of stroke patients, the time from admission to fibrinolysis, as well as the number of patients with a poor vital prognosis, was reduced. All of these studies show real benefit in patient management.

The review of the literature does not show a consensus on how to approach AVF or how to teach the procedure. Thus, we conducted a feasibility, monocentric, prospective, observational, before/after simulation training pilot study, SIMFAV (NCT03680209), at the University Hospital of Caen (unpublished data). We proposed a program with the help of nephrology nurses from a hemodialysis unit, nephrologists, a medical hypnosis practitioner, an ultrasound paramedic, and health simulation educators. We contend that the technical nature of the AVF puncture procedure must not overshadow health care providers’ relationship with the patient. Communication must be an integral part of technical care for the global care of the patient. Thus, the training program proposed in this study includes training in therapeutic communication with a hybrid simulation workshop (AVF puncture with a simulated patient). The results showed a decrease in complications (Kirkpatrick 4), such as a reduction in observed hematomas (*p* = 0.001) and a reduction in pain intensity (*p* = 0.03).

Therefore, we propose the SIMFAV2 study, a multicenter, randomized, comparative study between a group of providers that received only theoretical training and a group that received theoretical training and procedural simulation training.

## Objectives {7}

We hypothesize that a training program for nurses on AVF approach in procedural simulation versus theoretical input alone would decrease the adverse events related to AVF punctures and would be beneficial for the patient. As the patient’s management is not modified, only the consequences of the optimization of the procedure will be evaluated through the collection of the number of adverse events occurring during AVF puncture.

The primary objective was to compare the number of adverse events related to AVF puncture between a group receiving theory- and simulation-based training (TTST) and a group receiving only theoretical training (TT).

There were secondary objectives that were to compare the TTST and TT groups: occurrence of other adverse events related to AVF puncture during a hemodialysis session, patients’ experience of pain and anxiety, and nurses’ behavioral changes in medical device use.

## Trial design {8}

This is a prospective, multicenter cluster randomized controlled trial.

All centers use the same types of medical devices (catheters and puncture needles) and have an ultrasound machine available on site. The number of daily punctures varies from 13 to 40 depending on the center. A center effect will be sought.

Only two centers have a lower number of patients and punctures, but they are still comparable. We therefore propose to match the centers according to number of patients and punctures per day. We grouped the centers into pairs according to the criteria of number of AVF punctures per day, number of nurses, before randomization.

The different experimental and control groups will move forward in pairs (e.g., center A of the experimental group, center A of the control group).

**Fig. 1:**
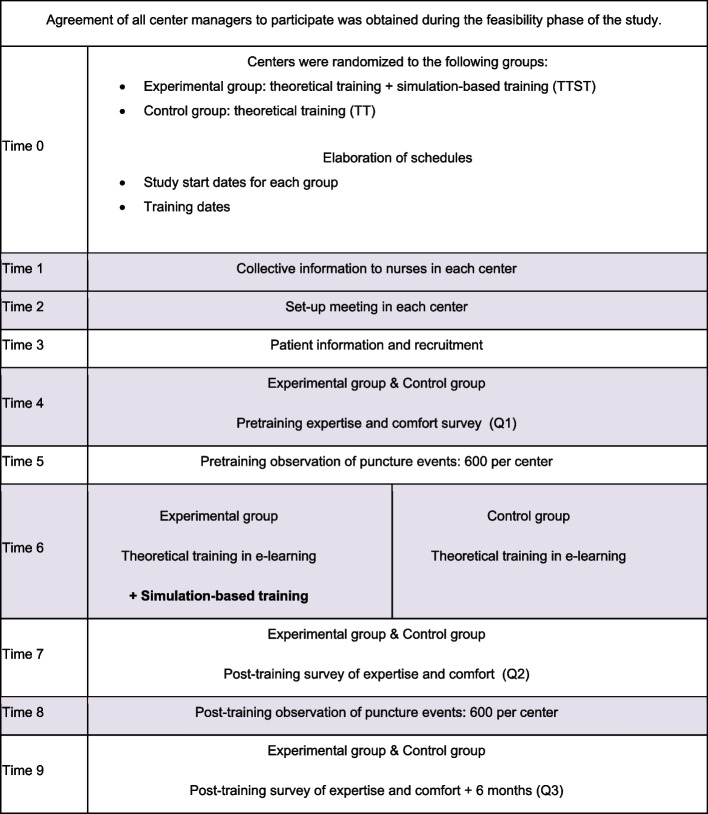
Trial design

The trial design is described in Fig. 1.

## Methods: participants, interventions, and outcomes

### Study setting {9}

This study involves 8 hemodialysis centers in a French district, the Normandy. We called for volunteers to take part in the study. Among participating centers, there are the community centers of Dieppe, Mémorial France Etats-Unis of Saint-Lô, Lisieux; the nonprofit centers “ANIDER” of Alençon, Hérouville Saint-Clair, Flers and Dieppe; and a private hospital Saint Martin of Caen.

There will be approximately 140 nurses participating in this study. They will complete a data collection on the occurrence of adverse events. An anonymity number will be assigned to each participating nurse.

The start of the study for each center will be staggered by 1 month to allow for the distribution of training.

Data are collected in each hemodialysis centers and then centralized in the Caen University Hospital.

### Eligibility criteria {10}

All patients aged over age 18 scheduled for AVF puncture by a hemodialysis-trained nurse during a scheduled chronic dialysis session were eligible.

All dialysis nurses puncturing the included patients will be included in the study.

#### Criteria for non-inclusion

Patients who met the following criteria were excluded: having an AVF puncture in the emergency unit, refusing data collection, not speaking French, being less than 18 years old, being under guardianship or unable to give informed consent, being enrolled in a social security plan, being pregnant, or breastfeeding.

### Who will take informed consent? {26a}

The coordinating investigator will give eligible participants a detailed oral and written description of the study. An opportunity to ask questions will be provided before written informed consent is obtained.

### Additional consent provisions for collection and use of participant data and biological specimens {26b}

N/A. There are no planned ancillary studies involving the collection of data for purposes separate from the main trial. No additional biological samples will be collected for use in future studies.

## Interventions

### Explanation for the choice of comparators {6b}

Currently, with the advent of the digital age, access to e-learning courses, sometimes free of charge, is easy. To evaluate the effectiveness and usefulness of the procedural simulation itself, we will compare two groups of professionals: one receiving theory training in e-learning and simulation-based training and a group receiving only theoretical training in e-learning.

### Intervention description {11a}

Interventions for each group with sufficient detail to allow replication, including how and when they will be administered.

### Control arm (TT)

This group of nurses will benefit from a theoretical distance learning program that is based on the following:

- Rules of hygiene, asepsis, and operator protection, in accordance with good practice [27, 28]
- Optimal approach to the AVF: spotting, palpation, examination
- Effective fixation of the catheter
- Basic principles of therapeutic communication
- Basic principles of ultrasound and operation of the ultrasound machine
- Ultrasound identification/guidance of the AVF

At the end of the training program, nurses will be evaluated with a minimum passing score of 90%. Otherwise, nurses have to repeat the program until they achieve a satisfactory score, according to the principle of “mastery learning”, within the time limit of 1 month after the start of the training. Immediate feedback will be provided.

### Experimental arm (TTST)

#### First phase: theoretical training

This group of nurses will receive the same theoretical distance learning program as the experimental group. A notification of the success of the evaluation will be sent by the trainer to the promotor to begin the posttraining data collection phase.

#### Second phase: simulation-based training

The training at the next center starts at the end of the training of the previous center (duration per center: approximately 2 months).

The simulation-based training program is based on the pedagogical strategies described in simulation and seems to be relevant in this context, i.e., deliberate practice, mastery learning, and rapid cycle deliberate practice.

Peyton’s “4 steps” method is used for new learning. We will propose a variation of this learning tool that is more adapted for a group.

A “minimum passing score” (MPS) will be established on the main steps necessary to perform the procedure, including respect for good hygiene practices and effective communication. If the MPS is not reached, the learner can repeat the different workshops until an acceptable score is obtained (90% of correct answers).

The training will take place on a procedural mannequin, that is, an arm with a pulsatile AVF that is echogenic.

The training also includes a scenario with simulated patients using the “rapid cycle deliberate practice” method [19]. It is relevant to work on communication with the patient during procedural simulation.

#### Simulation-based training program

The program is composed of one-day training sessions at the NorSimS simulation center, with a maximum of 4 to 6 nurses per session. It includes different modules:

- Module 1/AVF puncture: theoretical review, puncture on simulator with application (location, examination, puncture, needle fixation, compression post puncture)
- Module 2/therapeutic communication: with simulated patient
- Module 3/ultrasound: manipulation with echogenic patches and AVF arms, ultrasound identification/guidance
- Module 4/training restitution: This hybrid simulation consists of using a simulator for invasive procedures and a simulated patient for communication training

### Criteria for discontinuing or modifying allocated interventions {11b}

According to the French Law n° 2009–879 of July 21, 2009, on hospital reform, patients, health, and the territory, all health professionals are obliged to be trained. Thus, it has been agreed with the volunteer centers participating in this study that all nurses working in these services will participate in the training. However, patients can withdraw from the study at any time.

### Strategies to improve adherence to interventions {11c}

To ensure the motivation of the nurses in the control group, they will receive simulation-based training once all the data needed for the study have been collected.

Additionally, nurses are thanked for their participation in the whole study with a culture voucher of a total value of 100 euros. These vouchers will be distributed to the nurses who complete the entire study (pretraining, training, and posttraining survey).

### Relevant concomitant care permitted or prohibited during the trial {11d}

There is no prohibited concomitant care.

### Provisions for post-trial care {30}

There will be no provisions after this trial.

Due to interventional research type (low risk one), the sponsor took out an insurance policy specific for this study that will compensate if there is any injury attributed to the randomized control trial.

### Outcomes {12}

The primary outcome is the number of adverse events occurring during AVF puncture in the TT and TTST groups (comparison of percent occurrence of events in each group) defined by any of the following events: unipuncture failure, bipuncture failure, simple hematoma, hematoma not allowing continuation of dialysis.

The secondary outcomes are the number of perdialytic bleeds: accidental needle withdrawal, pain score (numerical scale) experience at the time of puncture, ease or difficulty of puncture, patients’ anxiety score (numerical scale) at the time of puncture, use of ultrasound by the nurse, and type of equipment used for the puncture (needle or catheter).

### Participant timeline {13}

**Fig. 2:**
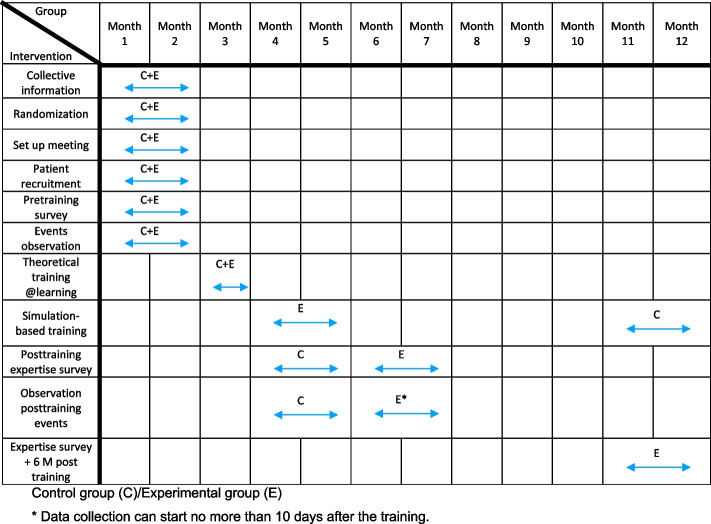
Participant timeline

The participant timeline is presented in Fig. 2

### Sample size {14}

This is a comparative, prospective, multicenter evaluation study taking place in 8 centers. The population is made up of all nurses performing hemodialysis punctures. We expect a reduction of at least 50% of the complications related to the puncture in the experimental group compared to the control group (possible result following the first pilot feasibility study, monocentric, prospective, observational, before/after, SIMFAV (RCT03680209) carried out at the University Hospital of Caen Normandy). To show a significant difference at T8, a minimum of 1 complication per day (the least favorable condition for confirming our hypothesis) was considered, i.e., over 4 weeks and for 8 centers, 224 complications in the control group, or 9.3%. For a reduction of at least 50% (4.65%), a risk *α* equal to 5%, and a power of 90%, it is necessary to have a total of 610 punctures, i.e., approximately 80 per institution. The randomization of hospitals and not of caregivers (registered nurse) allows us to avoid the risk of contamination between the two groups and to simplify the organization of the study within each institution. The Hawthorne effect should be reduced by the fact that, at the end of the study, the TT group will benefit in the same way as the TTST group from the procedural and hybrid simulation training. This should ensure that all the caregivers will adhere to the project and thus be motivated to do so in a perfectly equitable manner. The methodology of randomizing hospitals and not caregivers, the so-called cluster approach, leads to caregivers in the same cluster being more similar (intraclass/intracluster correlation, ICC) than caregivers from different institutions randomized globally. To correct for this impact on variance, it is necessary to apply a variance inflation correction factor equal to (1 + (*m*-1) *r*) to the required number of subjects, with *m* equal to the realized number of punctures within a cluster and *r* being the ICC. The value of *r* can be relatively high because of the way in which a facility or department performs its nursing practice. To be able to carry out all the analyses and to take into account additional analyses (center effect, effect of the number of punctures performed and the degree of experience of the nurse, etc.), and feasibility, the highest number of 600 punctures per center, i.e., 4800 punctures, 2400 per group, is used. This number of 600 punctures per center takes into account the number of establishments.

The same number of 4800 punctures for the T5 period (baseline) is also used. This means a total of 9600 punctures over the study period.

Several levels are taken into consideration: center, nurse, patient, and AVF puncture. Within a single center, the overall reflection of activity is related to history, practices, schedules, recommendations, etc. This center-specific activity may differ from one center to another. The experience of the nurses led to the same effects from one center to another, such as the characteristics of the patients and the AVF. These different points are taken into consideration to look for possible differences and effects that could be the cause of confounding factors.

In total, 8 centers participated, with 600 pretraining punctures per center and 600 posttraining punctures per center; that is, 9600 punctures were observed.

### Recruitment {15}

All patients received hemodialysis in the center, excluding those who needed emergency care. There are between 17 and 34 punctures per day. Target sample sizes are not difficult to achieve.

## Assignment of interventions: allocation

### Sequence generation {16a}

We propose to match the 8 centers in their characteristics of number of patients and punctures per day.

The different experimental and control groups move forward in pairs.

There are 4 pairs, each comprising two centers. They are constituted in such a way that the 2 centers in each pair have similar characteristics, such as a comparable level of activity, a similar number of caregivers, particularly nurses, and a similar geographical environment in terms of urban and rural characteristics.

**Fig. 3:**
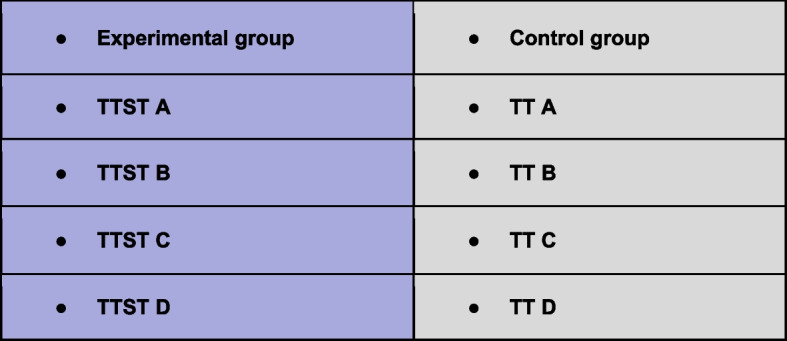
Randomization

There were 4 centralized randomizations carried out by the Clinical Research Department of the Caen University Hospital. The TT group and the TTST group for each pair are already constituted. Consent, contrary to the Zelen method, is requested before randomization (Fig. 3).

### Concealment mechanism {16b}

All center managers gave their agreement to participate before the project began. Arm allocation for each pair is generated by a computer.

### Implementation {16c}

The pairs associating two centers were constituted by taking into account the level of activity, i.e., the number of dialysis patients managed and the number of caregivers present. The allocation of a pair in the group to the experimental group (or not) was done by computerized random draw. The randomization was conducted by the Clinical Research Department of the University Hospital Center of Caen. Four centers were in the control group, and 4 centers were in the experimental group.

To organize the training for each pair of centers, randomization was conducted by the Clinical Research Department of the University Hospital Center of Caen. Four centers were in the control group, and 4 centers were in the experimental group.

## Assignment of interventions: blinding

### Who will be blinded {17a}

N/A. Blinding is not possible in this study.

### Procedure for unblinding if needed {17b}

N/A. Blinding is not possible in this study.

## Data collection and management

### Plans for assessment and collection of outcomes {18a}

Data will be managed in a database administered by the sponsor. Data entry will be performed by the investigators at each center and may be performed by any person on the task delegation list. The system used for the computerized database will be in accordance with the regulations in force, particularly MR-01. Access to the database will be limited to authorized persons only (sponsor team, investigation team, principal investigator), and rights (read, write, specific pages of the case report form, patients of the center or all patients, etc.) will be assigned according to their role in the study and their center. Authentication of users will be assured by means of a username and a password personalized for each user. Connections to the database will be recorded in the connection history.

Consistency tests may be scheduled depending on the level of risk and/or impact of the study as defined by the sponsor. Following the execution of these tests, requests for clarification (queries) may be sent to the investigators to correct or confirm data entered in the database. Depending on the level of risk and/or impact of the study, a preanalysis committee may meet before the data are exported to qualify the deviations identified during the study and to decide whether or not to include the data collected in the study.

### Plans to promote participant retention and complete follow-up {18b}

Data are collected in the software used routinely in an e-case report form with the software EnnovClinical©.

### Data management {19}

Database development and administration are ensured by the North-West Canceropole Data Processing Centre (DPC) and under Caen University Hospital responsibility. Access is allowed to authorized people only through a highly secured system (VPN/SSL mode up to 128 bits; transmitted data encrypted). Its web interface allows study management via standard data entry in client/server and/or via the internet. Daily backup is made using two independent servers.

Data protection is based on (i) the definition of authorization profiles, defining the functions or types of information available to a user; (ii) control of access to the software by a username issued by the administrator and a password; (iii) electronic signature of the data; (iv) secure access to data by the “owner” only; (v) the audit trail that tracks all access, modification, and deletion of data; (vi) exported files archived within the system; (vii) all plotted transfers and uploads and downloads; and (viii) the possibility of automatic identification of patients without nominative data (creation of a unique patient identification number (alphanumeric) to enable interoperability of the data recorded and preserve anonymity).

Data quality is assured by pretesting and consistency checks during data entry. The software also includes a data management system of “queries.”

Storage: The Ennov Clinical© application is made available in SaaS mode by ENNOV™, which outsources its server installation and hosting services (software and data) to OVH™.

Backups are made daily (databases and servers). This is an incremental backup without overwriting data. A history is created allowing, if necessary, the recovery of previous data.

Data management/quality: The data processing center has quality procedures relating to Good Clinical Practice (GCP) guidelines and the use of the software. The DPC has been certified by the French National Cancer Institute (INCa) since July 2007 and International Organization for Standardization 9001:2015 since March 2018.

### Confidentiality {27}

Data collection is anonymous.

A number will be assigned to the nurses. This number will be known only to them. Neither the investigators nor other nurses will be aware of it. It will prevent any potential feelings of judgment or individual evaluation.

### Plans for collection, laboratory evaluation, and storage of biological specimens for genetic or molecular analysis in this trial/future use {33}

N/A. No biological specimens obtained during the conduct of the trial will be stored for future use.

## Statistical methods

### Statistical methods for primary and secondary outcomes {20a}

The difference in percentages between the two groups will be determined by a chi-square test, thus allowing us to meet the main objective. This result will be adjusted according to several criteria, two of them in particular: a center effect, a baseline effect, and an effect of the degree of experience of nurses.

Indeed, the analysis of cluster trials is more complex than the analysis of trials with individual randomization units. The analysis will therefore also be carried out at the cluster level using the guide of the French Health Authority of June 2007 (methodological guide downloadable from www.has-sante.fr).

The secondary objectives (number of per-dialytic bleeds, accidental needle withdrawals) will be analyzed according to the same procedures as for the primary objective. The pain score (numeric scale) at the time of puncture will be analyzed between the two groups by Student’s *t* test.

All the parameters collected will be analyzed.

Multivariate analyses will be considered according to the results of univariate analyses such as logistic regression comparing the two groups and mixed models.

The adjustments described above will also be applied to the secondary objectives.

The collection of the nurses’ feelings of expertise will be synthesized.

All analyses will be based on a first species error of 5% and will be two-tailed.

The IBM®-SPSS® data analysis software (IBM Corp. Released 2015. IBM SPSS Statistics for Windows, Version 23.0. Armonk, NY: IBM Corp.) will support all analyses.

### Interim analyses {21b}

N/A. No interim analyses are planned.

### Methods for additional analyses (e.g., subgroup analyses) {20b}

The difference in percentages between the two groups will be determined by a chi-square test, thus allowing the main objective to be met. This result will be adjusted according to several criteria, in particular two of them: a center effect, a baseline effect, and an effect of the degree of experience of the nurse.

Indeed, the analysis of cluster trials is more complex than the analysis of trials with individual randomization units. The analysis will therefore also be performed at the cluster level using the Health French Authority of June 2007 cluster (methodological guide downloadable from www.has-sante.fr).

### Methods in analysis to handle protocol non-adherence and any statistical methods to handle missing data {20c}

For missing data, if less than 5% of the sample contains at least one missing data point, it will not be imputed. The analysis will be performed as a complete case analysis. If more than 5% of the sample contains at least one missing data point, there are two options. If a variable contains less than 20% of missing data, we perform multiple imputation by chained equations (MICE). If a variable contains more than 20% missing data, it cannot be introduced in a statistical model.

### Plans to give access to the full protocol, participant-level data and statistical code {31c}

There are no plans for granting public access to the full protocol, participant-level data, and statistical code.

The datasets analyzed during the current study and statistical code are available from the corresponding author on reasonable request, as is the full protocol.

## Oversight and monitoring

### Composition of the coordinating center and trial steering committee {5d}

No trial steering committee is defined in the protocol. The coordinating investigator and sponsor project manager provide day-to-day management of the trial. There is no Stakeholder and Public Involvement Group (SPIG).

### Composition of the data monitoring committee, its role and reporting structure {21a}

The medical procedures used in this trial comply with the most recent recommendations of the Declaration of Helsinki and French Public Health Law 2004–806 of August 9, 2004, on subject protection and safety in accordance with good clinical practice. A person mandated by the sponsor will ensure monitoring of this trial to guarantee that accurate, full, and reliable data are collected. The level of monitoring will be adapted to the low risk of the study.

At the end of the study, the data review committee, comprising the data manager, the biostatistician, and an independent dermatologist, will review all deviations from the protocol. Other members may join the committee to provide some details on the context of each deviation. The committee will qualify deviations as major or minor and shall clarify the relevance of the data with respect to these deviations: conservation of the data (for minor deviation) or exclusion of the data (for major deviation). Major deviations can affect subject safety or rights. Additionally, by definition, the intention-to-treat analysis requires that all data be kept for the analysis, even major deviations, except in the event of absence or withdrawal of written consent that systematically results in the exclusion of any data on the research.

### Adverse event reporting and harms {22}

No risks were identified for patients in this study. The adverse events observed are those usually seen in standard care. The primary outcome is the number of adverse events occurring during AVF puncture: unipuncture failure, bipuncture failure, simple hematoma, hematoma not allowing continuation of dialysis. The coordinating investigator will be informed by the study staff in case of any other adverse events. The coordinating investigator will notify the sponsor by email without delay.

### Frequency and plans for auditing trial conduct {23}

Frequency and procedures for auditing trial conduct, if any, and whether the process will be independent of investigators and the sponsor. No audits of this study are planned.

### Plans for communicating important protocol amendments to relevant parties (e.g., trial participants, ethical committees) {25}

When applicable, the trial steering committee will communicate substantial protocol modifications to relevant parties (People Protection Committee, trial participants and study professionals). The protocol in in the clinical trial registry will be updated.

### Dissemination plans {31a}

The results of the trial will be published in international peer-reviewed scientific journals. Participants, patients, and nurses will also be informed.

## Discussion

AVF puncture in patients undergoing chronic hemodialysis is a procedure that nurses repeat many times. The quality of the procedure determines the success of the treatment and the patient’s comfort. This procedure requires specific skills to avoid multiple complications.

Currently, the technique for puncturing AVF is passed by companionship within hemodialysis departments. Standardization of the procedure is part of a drive to optimize practices and continuously improve care. In this study, we expect to observe a significant reduction in pain intensity for patients, as well as a reduction in the number of hematomas. The reduction in these adverse events will lead to an extension of AVF lifespan, an increase in dialysis quality, and possibly a gain in quality of life for the patient.

The strong point of our study is the use of procedural training, which is innovative in several aspects. It is little used in the continuing education of professionals. It enables the team approach to AVF puncture to be conceptualized and then modeled in procedural simulation. The contribution of therapeutic communication in procedural workshops enables a holistic approach to patient management, despite the technical nature of the treatment. Moreover, the evaluation of the direct benefit of training for the patient (Kirkpatrick level 4) is poorly described in the literature.

This study has several limitations. Firstly, data collection concerning the occurrence of adverse events is declarative in nature. Nurses may underreport/overreport the occurrence of events for fear of being judged. This risk is present in both the data collection and the comfort questionnaire. Then, with regard to distance learning, we cannot be totally sure that it is the nurses who log on, carry out the distance learning, and validate the quizzes.

## Trial status

The protocol version number is V03_30.06.2022. Recruitment began in September 2022, and we expect recruitment to be completed by March 2024.

## Data Availability

The principal coordinating investigator, the monitoring team, and the methodologist will have access to the final dataset. Dataset is under sponsor responsibility, and any data required to support the protocol can be supplied on request directly with the sponsor.

## Abbreviations

CRF: Case report form
NS: Numeric scale
AVF: Arteriovenous fistula
HD: Hemodialysis
TT: Theoretical training group (control group)
TTST: Theoretical training + simulation-based training group (experimental group)
